# Exploring user-generated content related to vegetarian customers in restaurants: An analysis of online reviews

**DOI:** 10.3389/fpsyg.2022.1043844

**Published:** 2023-01-10

**Authors:** Shizhen Bai, Xuezhen Zheng, Chunjia Han, Xinrui Bi

**Affiliations:** ^1^School of Management, Harbin University of Commerce, Harbin, China; ^2^Department of Management, Birkbeck, University of London, London, United Kingdom

**Keywords:** vegetarian customers, dining experience, user-generated content (UGC), word embedding, topic modeling, restaurant review

## Abstract

This study aimed to explore and evaluate factors that impact the dining experience of vegetarian consumers within a range of vegetarian-friendly restaurants. To explore the factors and understand consumer experience, this study analyzed a vast number of user-generated contents of vegetarian consumers, which have become vital sources of consumer experience information. This study utilized machine-learning techniques and traditional methods to examine 54,299 TripAdvisor reviews of approximately 1,008 vegetarian-friendly restaurants in London. The study identified 21 topics that represent a holistic opinion influencing the dining experience of vegetarian customers. The results suggested that “value” is the most popular topic and had the highest topic percentage. The results of regression analyses revealed that five topics had a significant impact on restaurant ratings, while 12 topics had negative impacts. Restaurant managers who pay close attention to vegetarian aspects may utilize the findings of this study to satisfy vegetarian consumer requirements better and enhance service operations.

## 1. Introduction

Due to the impact of dietary options on health concerns and environmental sustainability obtaining increasing attention, the number of vegetarians and the consumption of vegetarian food are continuously increasing in parallel (Contini et al., 2020; Kamiński et al., 2020). Vegetarian diets are gradually being perceived as an appealing lifestyle that can provide health benefits (Craig, 2009; Le and Sabaté, 2014; Faber et al., 2019). Many studies have shown that vegetarian foods can provide sufficient nutrition and effectively prevent specific cardiovascular diseases, diabetes, and cancers (Kahleova et al., 2017; Wells, 2019) and even reduce the risk of death (Springmann et al., 2016). In addition, the meat production process causes environmental issues, as well as a consciousness of abating meat consumption and concerning animal welfare, are consistent for many consumers (Ghahari and McAdam, 2018; Chai et al., 2019; Grossmann and McClements, 2021). Nowadays, the popularity of vegetarianism has brought new opportunities to some fields, especially the food and beverage industry. A Bloomberg 2021 report predicts that the global market of plant-based alternatives may grow from $29.4 billion in 2020 to $162 billion in the next decade (Bartashus and Srinivasan, 2021). In addition, the variety of vegetarian foods available is also gradually diversified into beverages and animal-analog food products, such as plant-based meat, fish, eggs, milk, and cheese (He et al., 2020; McClements et al., 2021).

Although dining out has become a popular and prominent social activity for consumers around the world (National Restaurant Association, 2018), it is challenging for vegetarian customers due to the particularity of their diet. A study by VEGANUARY in 2022 found that 26% of participants thought dining out was the biggest challenge during their Veganuary (The Veganuary, 2022). Many restaurants also suffer some financial losses due to their failure to cater to vegetarian customers. While aiming to meet the considerable demands of vegetarians and non-vegetarians who prefer to eat vegetarian food, many mainstream chain restaurants offer labeled vegetarian options on their menu, and some even prepare a separate vegetarian menu, but merely offering vegetarian menu options cannot guarantee the improvement of the vegetarian customer experience, which is a significant measure of competitive advantage in the restaurant industry [Hayeon (Hailey) Choi et al., 2021]. Restauranteurs must also be very familiar with the behavior, psychology, and requirements of vegetarians and fulfill them accordingly. Despite research into the value of vegetarian dietary requirements in London, Birmingham, and Manchester (Mathayomchan and Taecharungroj, 2020) and studies on the attitudes toward supplying vegetarian options by restaurants in Puerto Rican (Rivera and Shani, 2013), this dietary trend has not been extensively studied, particularly in terms of customer experience.

Considering that customers have voiced an increasing enthusiasm to eat vegetarian foods (Choi and Joung, 2018) and the benefits of accommodating this customer group, researchers have been executing studies looking into the challenges of catering to vegetarian customers (Pohjolainen et al., 2015; Oh et al., 2021). These studies have mostly applied research methods, including individual interviews, questionnaires, and online surveys. These traditional quantitative and qualitative approaches have deficiencies such as limited generalizability because of both insufficient samples and a lack of comprehension of vegetarian customers’ perceptions due to the nature of the presupposed conditions (Wen et al., 2019). Furthermore, based on the development of big data technology, customers will browse online restaurants’ menu options, ratings, and other customers’ reviews when they select a restaurant (Yan et al., 2013). The massive number of online customer reviews not only offers rich information but also embodies customers’ authentic dining experiences across a wide range of contexts. However, few studies have been implemented focusing on user-generated content about the dining experience of vegetarian customers due to their extensive size and unstructured text formation (Kwon et al., 2020). Therefore, the current study aims to explore the main factors in customer reviews that are significant to vegetarian customers. In addition, although big data analytics have been utilized in customer studies in other fields, few studies have applied these methods to analyze vegetarian customers’ dining experiences with restaurants. The demands of vegetarian customers may be distinctive compared with those of other customers, and such a study would be extremely worthwhile (Wen et al., 2019).

Therefore, the purpose of this study was to explore and evaluate factors impacting the dining experience of vegetarian consumers within a range of vegetarian-friendly restaurants when fulfilling vegetarians’ demands by utilizing big data analyses and traditional methods (e.g., regression analysis). The specific intentions were to (1) explore prevalent topics discussed by vegetarian customers on TripAdvisor.com; (2) identify factors involving the satisfaction and dissatisfaction of vegetarian customers; (3) evaluate the relative significance of satisfaction and dissatisfaction factors on restaurant ratings; and (4) make recommendations and suggestions to restaurant managers to satisfy vegetarian customers.

The remainder of this study is summarized as follows. In section “Literature review,” the literature review is introduced. In section “Methodology,” the methodological process is presented. In section “Results and discussion,” the results and discussions are provided. Finally, we conclude with implications from the analysis and suggestions for future research in the “Conclusion” section.

## 2. Literature review

### 2.1. Vegetarianism and vegetarians

Vegetarianism is usually described as a dietary pattern featured by the avoidance of some or all animal-based foodstuffs and the consumption of plant-based foods (Perry et al., 2001, p. 406). Although it is generally believed that vegetarians are a homogeneous group, they have many forms and types. The most convenient way to distinguish vegetarians is according to the food categories they choose to include in or omit from their diet, including semi-vegetarian/flexitarian (consumes vegetarian most of the time, occasionally meat), pesco-vegetarian (consumes seafood), pollo-vegetarian (consumes poultry), lacto-ovo vegetarian (consumes dairy and eggs), lacto-vegetarian (consumes dairy), ovo-vegetarian (consumes eggs), and vegan (does not consume any animal-based products) (Beardsworth and Keil, 1991; Raphaely and Marinova, 2014). Many researchers have proposed that the general reasons for selecting a vegetarian diet are health concerns, ethical reasons, the welfare of animals, concerns about the environment, religious faith, social concerns, weight management, flavor, and abomination toward the sensory properties of meat (Ruby, 2012; Hoffman et al., 2013; Cramer et al., 2017; Plante et al., 2019; Šmugović et al., 2021). Whatever the classification basis may be, the vegetarian population has become far more varied and complex than may originally have been conceived.

In addition to the classification and motivation of vegetarians, the existing literature has also conducted much research on other aspects of vegetarianism. First, a large number of previous studies have examined the relationship between moral values and vegetarianism (Hayley et al., 2015; see De Backer and Hudders, 2015; Veser et al., 2015; Wrenn, 2017; Heiss and Hormes, 2018; Pfeiler and Egloff, 2018). Second, the barriers to becoming a vegetarian have been extensively studied. These researches reveal that perceived barriers to adopting a plant-based diet outweigh the perceived benefits, making them resistant to eating less meat (see Lea and Worsley, 2003; Lea et al., 2006; Ensaff et al., 2015; Corrin and Papadopoulos, 2017; Kildal and Syse, 2017; Forestell and Nezlek, 2018). Finally, existing studies have shown a link between vegetarianism and social implications, such as gender (see Ruby and Heine, 2011; Rozin et al., 2012; De Boer et al., 2017; Love and Sulikowski, 2018), identity (see Fox and Ward, 2008; Romo and Donovan-Kicken, 2012; Rosenfeld and Burrow, 2018), social experience (see Hirschler, 2011; MacInnis and Hodson, 2017), and culture (see Ruby and Heine, 2012; Sobal et al., 2014; Earle and Hodson, 2017).

### 2.2. The influence of vegetarianism on the restaurant industry

As the number of vegetarians in the world continues to grow exponentially, so does the number of vegetarian-friendly restaurants, which are restaurants that do not provide animal-based products in their food and beverages (The Beveg, 2021); that is, some or all menu options are meat- and dairy-free, and no animals, animal byproducts, or derivatives are utilized in the kitchen. At the end of 2019, Europe had slightly over 2,600 vegan restaurants listed, and by early 2022, the restaurant count had climbed to 3,400. That is a 25% jump (The Happycow, 2022).

In recent years, the growing requirements for vegetarian foods and the rising appeal of a healthy diet have brought about an increase in the variety and quality of plant-based food in many restaurants (Yee, 2004). Deliveroo had more than 14,000 vegan and vegan-friendly restaurants that were available for orders from its app by November 2021. Wagamama’s menu options were 50% plant-based by October 2021 (The Vegan foodandliving, 2020). Moreover, vegetarian food choices also can be affected by restaurant menu design (Bacon and Krpan, 2018). Using a separate menu that only includes vegetarian options could significantly improve the proportion of people choosing vegetarian food (Campbell-Arvai et al., 2012). Adding words related to enjoyment to the name of dishes could increase the choice of vegetarians (Turnwald et al., 2019). Redesigning the framework of vegetarian food names could improve the likelihood of vegetarian selection compared to an independent vegetarian frame in the restaurant menu (Krpan and Houtsma, 2020). In addition, the label of vegetarian options, which have preferred being showed by “vegetarian” or “vegan” labels on menus, packaging, and signs, can also affect customers’ choice of vegetarian food (Vlaeminck et al., 2014; Tobi et al., 2019). The study found that menu items explicitly indicating “vegetarian,” “vegan,” or “meat-free” reduced the number of diners willing to consume these foods. Although many restaurants are actively responding to this trend in various ways, including adding vegetarian items and designing and labeling the menu, it is still difficult to find ample vegetarian items among the menu options. An important reason seems to be the common view of vegetarian food as unattractive, strenuous, and dull and the trepidation about not getting enough nutrition from a vegetarian diet, which most chefs run away (Rowe, 2010). Therefore, many restaurants specifically recruit chefs for vegetarians because the preparation of high-quality vegetarian meals requires hard work and wide knowledge (Kuhn, 2006). Other limitations include having a limited and non-creative diversity of meatless options, a lack of professional knowledge among servers regarding menu options that are appropriative for vegetarians, no indication in menus regarding non-vegetarian food ingredients that can be turned into vegetarian items and situations in which meat or other animal ingredients are discovered in what was supposed to be vegetarians’ dishes (Yoo et al., 2022). The result is those vegetarian customers are limited when they go out to dinner (Perlik, 2010). Therefore, restaurant revenues may be negatively impacted, and non-vegetarian customers may be lost due to not fulfilling the demands of vegetarians (Rivera and Shani, 2013).

### 2.3. User-generated content in the restaurant industry

Social media and websites are convenient online platforms that enable users to share and communicate their opinions, which help present customers’ experiences, voice their perceptions and emotions, and provide recommendations and suggestions for products or services (Lu and Stepchenkova, 2014; Nilashi et al., 2019). Online reviews and ratings generated from these platforms constitute two of the most significant forms of user-generated content (UGC) (Ye et al., 2011). When there are both review text and review ratings, customers tend to make efforts to deal with these two forms of information to make more accurate and feasible decisions (Mudambi et al., 2014). In particular, online reviews have a large impact on consumers’ behavior and intention and have become an important source for tourism, restaurant, hospitality, and academic research because they provide more detailed and comprehensive customer feedback (Guo et al., 2017).

Due to the fact that most customers usually utilize online platforms to search for information before deciding which restaurant to visit, user-generated content is becoming increasingly important for them (Yan et al., 2013). Generally, online review releases from consumers express either a positive or negative description of a restaurant (Banerjee and Chua, 2016), and customers conversely believe in and respond to such signals by behaving either positively or negatively toward the restaurants. For example, previous research has shown that the number of reviews has a positive influence on the profit and client counts of restaurants (Kim et al., 2016). Huifeng et al. (2020) studied the relevance between online reviews and customer revisits, indicating the effect gradually decreases over time. Higher user ratings can improve customers’ willingness to select take-out or dine-in restaurants (Ha et al., 2016). Higher ratings can also help restaurants sell out their tables more frequently. In addition, some scholars utilize user-generated content to explore issues related to the customer dining experience. Wen et al. (2019) explored and interpreted factors that impact the dining experience of customers with food allergies. Le et al. (2021) explored authenticity dimensions that are of value to customers in dining experiences. They found that “deviated authenticity,” which is a new type of authenticity compared to categorical and historical authenticity that have been explored in the previous literature, emerges as a second-level dimension falling under *Authenticity of the Other*. Huang (2017) compared the dining experiences of domestic and foreign English-speaking customers at Beijing Roast Duck. Researchers have also applied user-generated content to interpret factors affecting customer satisfaction or dissatisfaction. Wang et al. (2018) identified six emotions embedded in online reviews containing anger, fear, joy, trust, disgust, and sadness, which could be considered indicators of customer satisfaction or dissatisfaction. Jia (2019) found and compared the satisfaction of restaurant tourist customers traveling in four Nordic countries by analyzing their online reviews and ratings.

Because of the outbreak of COVID-19, pandemic-related issues change customers’ demands and preferences that determine their satisfaction, especially for the hygiene, and safety of restaurants. However, uncertainty about the security of dining out during crises can be relieved by browsing and sharing information about restaurants’ hygiene protocols and precautions on online review platforms that provide the latest and unfiltered information (Schroeder et al., 2013; Kim et al., 2021). Although customers’ preferences concerning food, service, and atmosphere will remain important and prominent in the long term, scholars will still make efforts to explore and explain new varieties of restaurant customers’ demands and preferences due to the outbreak of the COVID-19 pandemic (Luo and Xu, 2021). According to the previous study, restaurant customers had different responses to services before and during the pandemic (Chang et al., 2022). Based on multiple correspondence analysis and keywords analysis, Kostromitina et al. (2021) utilized 6,782 reviews during the COVID-19 pandemic and 2,145 reviews before the crisis from Yelp.com to explore criteria used by customers in allocating star ratings. Customers shift their review patterns, which tend to give lower star ratings, and evaluate the characteristics of the same restaurant in different ways. Jia (2021) indicated that customers become calm when they are in line, a situation that causes a negative influence in normal periods. Cao et al. (2021) extracted 3.1 million online reviews, utilized methods of sentiment analysis and topic modeling, and discovered that more than 10% of online reviews included COVID-associated keywords and that the prevalence of COVID-associated factors (e.g., mask, social distancing, and hygiene) raised during the COVID-19 pandemic, while the prevalence of other factors decreased. Luo and Xu (2021) analyzed 112,412 Yelp reviews posted during the crisis by applying methods of sentiment analysis and keyword analysis. They show that some keywords are utilized more frequently, such as “outdoor seating” and “hygiene precautions” related to dine-in experiences, and “online ordering,” “delivery,” and “UberEATS” in the field of take-out, compared to previous user reviews. On the other hand, Sun et al. (2022) discovered that restaurant customers were more inclined to offer higher star ratings and positive reviews after the crisis if the service staff had strictly complied with safety precautions and hygiene protocols.

### 2.4. Studies on text mining with big data analysis techniques

In the last decade, unstructured user-generated content grows exponentially and has exhibited great advantages in obtaining valuable information over traditional questionnaires. However, traditional manual content analysis becomes difficult and expensive, while big data analysis emerges at a historic moment and gains wide attention, especially in the hospitality and tourism industries. Machine-learning and deep learning methods have become powerful tools for efficient computing and intelligent analysis of online reviews, which allow the system to automatically learn from data and improve from experience without explicit programing (Van Calster, 2019).

The word embedding technique (Mikolov et al., 2013) is a machine-learning algorithm used to detect semantic similarity. This technique has received widespread attention from researchers due to its simplicity, credibility, and high processing speed (Fan et al., 2017). It is based on the distributional hypothesis that words appearing in the same context should have similar meanings. Word embedding is a low-dimensional vector representation of a word (generally used dimensions can be tens to thousands) (Evans and Aceves, 2016). After having a word vector, various vector-based calculations can be implemented, such as using the similarity between vectors to measure the semantic relatedness between words. However, few management researchers applied word embedding techniques. Zhang et al. (2015) inspected the semantic relationships and characteristics of words in online customer reviews utilizing word embedding. Yoo et al. (2022) investigated vegan and vegetarian subreddits to classify consumers’ interests and preferences.

Another famous machine-learning technique is topic modeling (Blei et al., 2003), which has been applied by researchers to find potential topics based on the co-occurrences of words in an extensive volume of online reviews (Kaplan and Vakili, 2014). This approach makes researchers quantitatively evaluate the importance of topics for each document. A few studies in tourism and hospitality management have applied topic modeling methods to identify market information that embodies consumers’ behaviors and perceptions over the past 40 years. For example, Kwon et al. (2020) explored the underlying factors of customer value through the topic modeling of UGC on Yelp.com. Wen et al. (2019) applied topic modeling to explore and evaluate factors impacting the perceptions of consumers with food allergies toward restaurants. Park et al. (2018) explored the diversification of research topics in hospitality in recent years and utilized time as a covariate in the model.

## 3. Methodology

**FIGURE 1:**
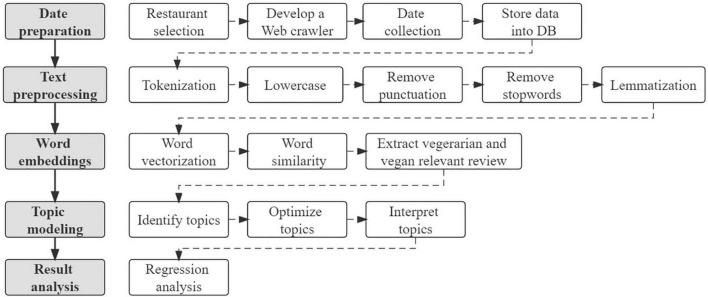
Methodological framework.

The methodological procedure of this current study consists of five key steps: Data preparation, text preprocessing, word embedding, topic modeling, and result analysis. The detailed phrases are shown in Figure 1. The following sections exhaustively explain these steps and the results.

### 3.1. Data preparation

The first step of data collection was the choice of vegetarian-friendly restaurants for this study. The current study collected online user-generated content of vegetarian-friendly restaurants from the TripAdvisor social website developed for hospitality and tourism service providers through a customized web crawler. First, the number of vegetarians and vegans in the UK quadrupled from 2014 to 2019 (Food Standards Agency, 2021), and it was the most popular country for vegetarianism in 2021, according to Google Trends (The Vegan Society, 2022). London has an increasing number of vegetarians and is also one of the gathering places for vegetarian-friendly restaurants. Second, TripAdvisor includes more than 1 billion reviews and opinions of nearly 8 million businesses and is regarded as a comprehensive platform for restaurant, hotel, and accommodation booking. Travelers and natives can utilize this platform to share their experiences and opinions on hotel and restaurant services; thus, the authors consider it a better data source. Finally, this research selected 1,008 vegetarian-friendly restaurants with more than 500 reviews in London. The period of the collected data was from January 2010 to May 2022, and the total amount of data is 1.04 million reviews, including restaurant names, URLs, descriptions, star ratings, review texts, review dates, and so on. The collected reviews were screened using two criteria: (1) Empty comments and (2) duplicate comments.

### 3.2. Data processing

Given that the extensive amount of user-generated content on online social platforms is highly unstructured, all data cannot be directly analyzed. Therefore, before implementing word embedding and topic modeling, text preprocessing provided by the Python library Spacy and NLTK was conducted to clean the dataset to guarantee better data quality. The steps of text preprocessing were as follows: (1) Removing non-English reviews and characters. There were multiple languages in the comments, such as English, French, Russian, and Chinese. Our study focuses on a single language, English, to ensure consistency among the texts analyzed; (2) removing stop-words, punctuation marks, and numbers; (3) transforming all texts into lowercase; and (4) lemmatizing and stemming. Through the above steps, all reviews were separated into minimally meaningful units called “tokens,” which were processed by a computer. The lemmatized tokens with a length of five characters or longer were extracted for data analysis.

### 3.3. Data analysis

#### 3.3.1. Word embedding

In this current study, the word embedding technique mainly captured the literal and even hidden implications of a target word, including vegan and vegetarian in a special context. This technique embeds many words in real-valued and continuous vector spaces where semantically similar words are mapped to adjacent points (Kwon et al., 2020). Since the crawled data are an unstructured natural language, word2vec is imported from the Gensim library for word embedding intentions. To search for semantically similar words, the word2vec algorithm utilizes cosine similarity, which calculates the cosine of the angle between two-word vectors to evaluate their similarity. It can effectively control magnitudes generated by frequent word occurrences in the vector spaces. All the reviews generated from the previous data preprocessing were trained to build a corpus. We used the skip-gram approach based on negative sampling to find word representations to predict the surrounding words in the text (Mikolov et al., 2013). For model training, the algorithm read five words before and after each target word and automatically moved forward to the next target word until the end of the word list. Moreover, the trained model established word embedding with 200 dimensions. We set the top 50 words that are similar to each target word and form a word vector including words and weight as the output vector.

#### 3.3.2. Structural topic modeling

Topic modeling is an unsupervised machine-learning method, and its foremost purpose is to reveal hidden structures (Blei, 2012) and all topics that are mentioned by customers about products or services in their reviews (Özdağoğlu et al., 2016). Among various topic modeling algorithms, structural topic modeling (STM) (Roberts et al., 2016) was employed to explore the significant factors influencing dining experiences and perceptions of restaurants’ ability to satisfy vegetarian customers because it permits researchers to incorporate arbitrary metadata with special topic proportions in each document to reveal a connection with any variable at the document level (Roberts et al., 2019).

**FIGURE 2:**
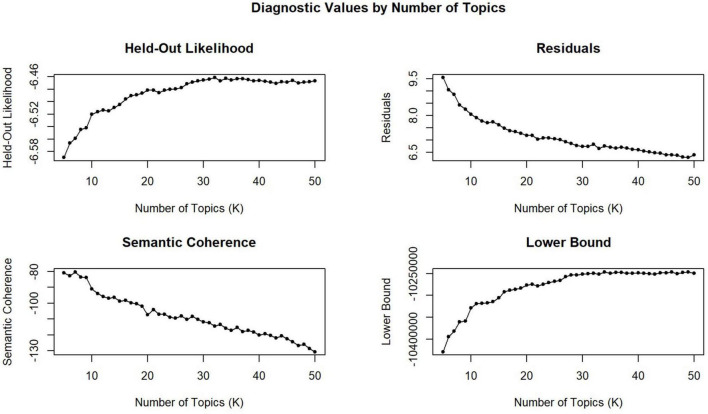
Diagnostic values by number of topics.

Based on final reviews, the STM algorithm was applied to process the data to identify topics or factors for topic modeling. To acquire the optimal number of topics (*k*), the current study established multiple models including different *k*-values (*k* = 5, 6, 7 … 49, 50) and compared the quantitative metrics, such as the held-out likelihood and semantic coherence. A model with 21 topics (Figure 2) was utilized based on the elbow range of the held-out likelihood, the medium-upper value of semantic coherence. With 21 topics, a probabilistic distribution of words over each topic and the document topic proportions were generated in light of the STM protocol. The top 15 words (e.g., Highest Prob and FREX) and the top 20 documents that were closest to each topic were examined to assess notable factors and create fitted topic labels (see Supplementary material for details).

#### 3.3.3. Multiple regression

After topic modeling, all reviews gained the statistical probability of topics, which were also called factors. The topic probability per review was utilized as an explanatory variable, and customers’ actual ratings of each review were regarded as a proxy for customer satisfaction. Multiple regression analysis was conducted utilizing SPSS (version 26.0) to estimate the relationship between identified factors and consumers’ ratings of restaurants.

## 4. Results and discussion

### 4.1. Words with semantic similarity to vegan and vegetarian

For this current study, the word2vec algorithm was applied to train the model, which comprised 71,724 words with 200 dimensions. Consequently, for any of the 71,724 words, the algorithm could discover 200 of the most similar words sorted by cosine similarity. Cosine similarity demonstrates how words are semantically similar to the target word (e.g., vegan and vegetarian), in which one in cosine similarity weight indicates that two-words are the same and zero implies that two-words have no semantic similarity. We set the target words as vegan and vegetarian in this study. For the target words “vegan” and “vegetarian,” the top 50 semantically similar words were detected and extracted, while the words with lower similarity scores were abandoned because the distance between them and the target word was relatively larger. Table 1 displays the semantically similar words and their weights. According to the outcome in Table 1, the vegan-related words included “vegetarian,” “veggie,” “gluten,” and so on, while the vegetarian-related word sets consisted of “veggie,” “vegan,” “non-meat,” and so on.

**TABLE 1:** Top 50 words with high cosine similarity to vegan and vegetarian.

	Word
Vegan	Vegetarian (0.879), veggie (0.815), gluten (0.771), non-vegan (0.764), glutenfree (0.744), coeliac (0.742), non-vegans (0.725), non-gluten (0.723), dairy-free (0.722), omnivore (0.721), lacto (0.720), pescatarians (0.718), pescatarian (0.714), dairy (0.712), coeliacs (0.705), pescetarian (0.704), non-veggies (0.702), gf (0.702), intolerant (0.698), glutenintolerant (0.696), meateaters (0.695), lactose (0.693), ceoliac (0.691), meatfree (0.690), non-meat (0.689), vegi (0.685), vegetarian (0.685), pescetarians (0.684), celiacs (0.683), vegeterians (0.681), meateater (0.679), plant-based (0.677), veganuary (0.677), non-vegetarian (0.675), kosher (0.675), vege (0.674), gluton (0.672), meatlovers (0.671), df (0.671), carnivore (0.669), veggie (0.668), pescatarian (0.668), vegetarian (0.667), celiac (0.664), non-seafood (0.662), meateating (0.662), paleo (0.660), herbivore (0.658), nutfree (0.658), non-veggie (0.657)
Vegetarian	Veggie (0.893), vegan (0.879), non-meat (0.779), omnivore (0.769), pescatarian (0.768), non-vegetarian (0.764), non-veggies (0.761), pescatarians (0.759), meateaters (0.750), vege (0.739), meatfree (0.739), non-fish (0.734), pescetarian (0.733), vegetarian (0.731), non-veggie (0.727), veggie (0.719), non-seafood (0.718), pescetarians (0.717), carnivore (0.717), vegi (0.716), vegeterian (0.712), meateater (0.710), herbivore (0.708), veggy (0.707), non-vegan (0.706), non-vegans (0.704), non-gluten (0.704), meatlovers (0.703), vegeterians (0.697), vegetarian (0.695), meateating (0.684), non-vegetarians (0.683), lacto (0.680), kosher (0.666),omnivorous (0.665), eater (0.664), omni (0.662), meatbased (0.660), gluten (0.660), vegitarians (0.658), glutenintolerant (0.656), vegatarians (0.651), coeliac (0.651), pescatarian (0.647), non-sushi (0.647), non-veg (0.647), plant-based (0.643), veganuary (0.643), glutenfree (0.64*3*), vegie (0.643)

We removed the duplicate values in 100 words, and the rest of the words were utilized to extract more relevant comments to minimize noise from less or no relevant comments and promote the accuracy of results and processing speed. Finally, the study applied the 64 identified words to extract final comments from the raw dataset for the next phase. As a result, a total of 54,299 comments collected from 1,008 restaurants were eventually selected for topic modeling. Table 2 summarizes the final comment data. All restaurants have a rating of three or above. Most restaurants have positive ratings, between 4.0 and 4.5 (83.9% for restaurants).

**TABLE 2:** Sample profile.

Type of restaurants	*N*	%
Independently owned	954	94.6%
Chain	54	5.4%
**Ratings**		
3.0	14	1.4%
3.5	124	12.3%
4.0	486	48.2%
4.5	360	35.7%
5	24	2.4%
Total	1,008	100%

### 4.2. Topic distribution

**FIGURE 3:**
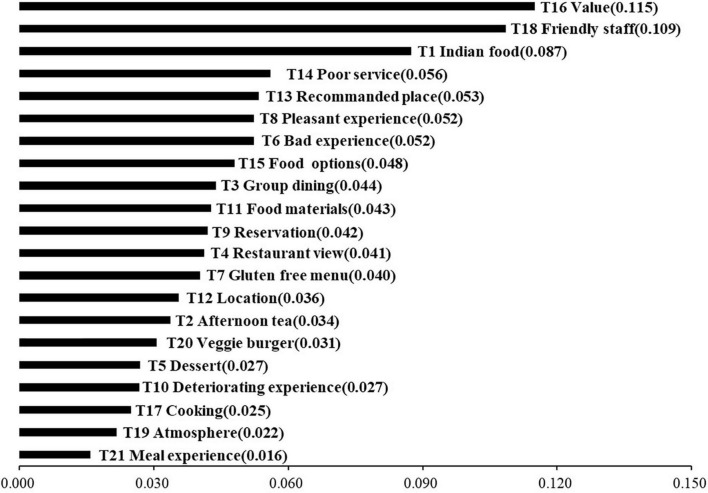
A total of 21 identified topics by prevalence.

Figure 3 illustrates the proportion of the 21 topics derived from the dataset.

Topic 16 (Value) had the highest topic proportion, accounting for 11.5% of the total topic proportion (topic proportion: 0.115). Value refers to customers’ total evaluation of the utility gained in products or services (Baker et al., 1994); it includes multi-dimensional indexes such as functional, cognitive, emotional, and social dimensions (Sweeney and Soutar, 2001). Consumers’ high attention to the value indicated that they preferred the trade-off between what they obtained and what they sacrificed. The second most prevalent topic was Topic 18 (Friendly staff), which made up 10.9% of the topic proportion among the 21 topics (topic proportion: 0.109). Friendly staff not only indicate great service quality but also understand the heterogeneity of the vegetarian population, including the dietary requirements of different types of vegetarians. The quality of services is always vital because it indicates whether customers are satisfied or dissatisfied with the services delivered in a vegetarian-friendly restaurant. The attitude and professionalism of vegetarian restaurant staff, including courteousness, responsiveness, assurance, reliability, care, and empathy, are essential factors that impact vegetarian restaurant customers’ dining experiences. In total, vegetarian customers pay more attention to whether restaurant staff respect and accommodate their special dietary habits so they can have a pleasant dining experience. Topic 3 (Indian food) revealed that Indian restaurants were an absolute paradise for those looking for tempting vegetarian options. Most Indian restaurants specialize in vegetarian dishes because vegetarianism has a long history related to traditional beliefs, rights, and status and is a practice born in India (Caplan, 2008; Preece, 2008).

In addition to the highest proportion of topic 1 (Indian food), seven food-related topics were identified (i.e., “T15 Food options,” “T11 Food materials,” “T7 Gluten free menu,” “T2 Afternoon tea,” “T20 Veggie burger,” “T5 Dessert,” and “T21 Meal experience”). Three topics related to overall experience were identified, and they were one positive factor (i.e., “T8 Pleasant experience”) and two negative factors (i.e., “T6 Bad experience” and “T10 Deteriorating experience”). Topic 6 (Bad experience) and topic 10 (Deteriorating experience) were both related to negative emotions, but their underlying sentiments were distinct. Topic 6 pertained to short-time perceptions to give a negative evaluation, while topic 10 contained long-term emotions, meaning that many restaurants had changed their business strategies to follow the trend, especially the transformation of food, making them lose their original advantages. The result shows that the original dish characteristics should be retained when developing new food items. Two location-related factors (i.e., “T4 Restaurant view” and “T12 Location”) indicated that the location of restaurants was a factor considered by vegetarian customers.

Topic 14 (Poor service), which included attitude and actual behavior, was identified as a factor. Topic 3 (Group dining) indicated that when there were many people at dinner, they were more inclined to choose vegetarian-friendly restaurants because they were likely to consist of vegetarians and non-vegetarians. Topic 13 (Recommended place) indicated that customers tend to utilize the online social platform to share their opinion after consumption (Hajli, 2018; Wang et al., 2020), and consumers also browse the opinions of other customers before making a decision (Gao et al., 2018). Topic 9 (Reservation) was identified as a convenience-related service attribute. Topic 17 (Cooking) played an important role in the quality of food. This topic was identified to indicate that vegetarian customers have strict requirements on the production method of vegetarian food because of the peculiarities of vegetarian materials. Topic 19 (Atmosphere) was also identified as a factor and played an important role in affecting the experience of consumers (Kim and Kim, 2004; Heung and Gu, 2012).

### 4.3. Regression analysis

Based on the previous section, we obtained the probability distributions of each topic at a metalevel and the topic probability value per review, which could be utilized as an independent variable to inspect the association with the reviewers’ ratings (Vu et al., 2019). In this part, regression analyses were executed to understand the relationships between each topic and the probability of attaining a higher star rating (Table 3). Because of the question of multicollinearity, Topic 1 was dropped from the regression model. The significantly positive beta values indicate that customers tend to give high ratings to restaurants when they mention these topics. In contrast, the topics with significantly negative beta values demonstrate that customers who mention these topics tend to render low ratings (Wen et al., 2019).

**TABLE 3:** Regression results to predict restaurant ratings.

Label	Beta	Standard error	*T*-value	Signature
T16 value	-1.281	0.042	-30.523	***
T18 friendly staff	1.459	0.041	35.389	***
T14 poor service	-4.417	0.045	-98.889	***
T13 recommended place	-0.026	0.059	-0.445	
T8 pleasant experience	2.097	0.054	38.736	***
T6 bad experience	-5.784	0.061	-94.860	***
T15 food options	-0.871	0.062	-14.165	***
T3 group dining	-0.272	0.071	-3.808	***
T11 food materials	-0.511	0.053	-9.647	***
T9 reservation	1.204	0.072	16.809	***
T4 restaurant view	-1.273	0.067	-19.069	***
T7 gluten-free menu	-0.808	0.046	-17.642	***
T12 location	-0.113	0.069	-1.643	
T2 afternoon tea	-2.223	0.053	-42.270	***
T20 veggie burger	-1.233	0.059	-20.900	***
T5 dessert	0.062	0.072	0.873	
T10 deteriorating experience	-6.305	0.071	-88.529	***
T17 cooking	-2.269	0.061	-37.469	***
T19 atmosphere	0.766	0.090	8.535	***
T21 meal experience	20.946	0.691	30.327	***

Reviews, *N* = 43,119; restaurants, *n* = 1,008; *** *p* < 0.001, *R*^2^ = 0.654.

As shown in Table 3, most of the correlations were statistically significant (*p* < 0.001), except for “T13 Recommended place,” “T12 Location,” and “T5 Dessert.” The topics that had positive associations with the ratings were less than the topics that had negative associations. Five topics had positive regression coefficients, indicating that customers were satisfied when they mentioned these topics. For example, “T18, Friendly staff” was positively correlated with customer satisfaction (beta = 1.459), and the other positive topics included “T8 Pleasant experience” (beta = 2.097), “T9 Reservation” (beta = 1.204), “T19 Atmosphere” (beta = 0.766), and “T21 Meal experience” (beta = 20.946). The results may show that, for vegetarian consumers, friendly staff, a relaxed atmosphere, providing reservation service, or serving delicious food may result in a pleasant dining experience.

On the contrary, 12 topics had negative associations with the ratings, revealing that if these factors are involved, customers are dissatisfied. First, “T10 Deteriorating experience” (beta = −6.305) and “T6 Bad experience” (beta = −5.784) had the strongest negative association with customer ratings. The result reinforces previous studies that customer experience is regarded as an important source of competitive advantage in the restaurant industry (Jeong and Jang, 2011; Ponnam and Balaji, 2014); a negative customer experience will lead to customer dissatisfaction and produce negative cognitive, emotional, and behavioral responses (Walter et al., 2010; Abdelhamied, 2011). Second, “T14 Poor service” (beta = −4.417) had a negative association with the ratings. This result is consistent with existing research, which emphasizes the importance of service quality in influencing customer satisfaction (Hwang and Ok, 2013; Tian et al., 2020). Third, “T17 Cooking” (beta = −2.269) was negatively correlated with the ratings. The results may indicate that many restaurants fail to properly cook vegetarian food for consumers due to the particularity of the materials. For restaurant chefs, it is essential to be familiar with the characteristics and collocation of ingredients and improve their cooking skills. Fourth, “T16 Value” (beta = −1.281) showed that evaluation toward value negatively affects the probability of obtaining high scores (Mathayomchan and Taecharungroj, 2020), implying that the absence of price fairness may result in consumer dissatisfaction.

Other negative factors included “T2 Afternoon tea” (beta = −2.223), “T4 Restaurant view” (beta = −1.273), “T20 Veggie burger” (beta = −1.233), “T15 Food options” (beta = −0.871), “T7 Gluten free menu” (beta = −0.808), “T11 Food materials” (beta = −0.511), and “T3 Group dining” (beta = −0.272). The results show that most factors related to restaurant food have been found to have negative effects; therefore, restaurants may need to improve these aspects to provide delicious food and satisfactory dining experiences for vegetarian consumers.

## 5. Conclusion

### 5.1. Theoretical implications

First, this study aims to fill the gap in the current research by exploring user-generated content related to vegetarian customers in the restaurant industry in London. By applying big data analysis technology to analyze user-generated content, this research contributed to the current literature on the dining experience of vegetarian customers by identifying the main topics. Compared with previous studies that utilized questionnaires and interviews, the results of this study reveal more general opinions while precluding the selection bias related to small or convenient samples (Huff and Kruszewska, 2016; Roberts et al., 2016).

Second, because customers freely share their dining opinions after actually visiting restaurants and are not influenced by researchers, the data provided are more reliable, and the results of this current study also have universality and suffer less social desirability bias. Big data analysis technology (e.g., word2vec and topic modeling) is utilized to analyze user reviews to help researchers discover factors implied in the user-generated content without being disturbed by their prior cognition of the factors or assumptions in the literature (Grimmer, 2014). In addition, the effects of each topic discovered from the large dataset can be quantified and illustrated by topic proportions (Roberts et al., 2014). For example, this study finds that topics including value (T16), friendly staff (T18), and Indian food (T1) occupy a large proportion of topics in the current review data. These findings indicate the importance of value for money and restaurant staff for vegetarian customers. In addition, Indian food is the major representative of a vegetarian diet because it has to do with religion and beliefs in India. Other significant findings include experience-related and food-related factors, group dining, reservations, and cooking.

Third, based on big data analytics (e.g., word2vec and topic modeling) combined with traditional methods (e.g., regression analyses), this current research identifies the factors and their degree of variance influencing customer satisfaction or dissatisfaction in vegetarian-friendly restaurants when they go out to eat. In total, this study utilizes modern methodology to help researchers understand the factors discerned from a larger amount of user-generated content and to provide suggestions to the restaurant industries, healthcare staff, policymakers, and vegetarian advocates.

### 5.2. Practical implications

The current study finds some significant practical implications for the vegetarian restaurant industry in London and, likewise, for restaurants in other districts that supply vegetarian diets. Although restauranteurs need extra effort to fulfill vegetarian customers, restaurants that recognize the consumer ability of vegetarians, and the rising number of sim-vegetarians/flexitarians can gain a competitive advantage over other restaurants that are tardier at understanding the vegetarian trend (Rivera and Shani, 2013). Decision-makers in the restaurant industries (i.e., managers, chefs, and owners) can utilize the results of this study to improve their operations to promote vegetarian clients’ satisfaction.

The recommendations and suggestions for the vegetarian restaurant industry are mainly described from the following six aspects. First, set reasonable prices to make consumers value for money. The importance of value for money is emphasized by the findings of the current study that vegetarian consumers complain about the high price of restaurants. Restaurant managers or operators should reasonably evaluate the value of food and services to avoid consumers’ perception of unfair prices. Second, properly train and educate restaurant staff to improve service quality. Training and educating staff are aimed to cater to the requirements of vegetarian clients. The proper staff training should involve knowledge related to vegetarian types and their motivations for becoming vegetarians, and the results from it are important to realize the heterogeneity among the vegetarian population. In addition, the positive staff education could keep them friendly and honest to accommodate vegetarian customers without discrimination and prejudice, resulting in this group of customers being delighted to improve their dining experience. Third, increase vegetarian food options and improve cooking skills. The importance of food has been greatly emphasized in existing research (Yim et al., 2014; Rhee et al., 2016; Taylor et al., 2018). Restaurant operators should invent high-quality and diversified vegetarian dishes to convert vegetarian customers from those who “only consume side dishes” and are compelled to stick to “the bread and water option” to clients who can entirely enjoy the dining-out experience with “rightful meals.” In addition, cooking technology is the key factor determining food taste. Compared with meat, due to the particularity of vegetarian ingredients, vegetarian materials are difficult to make delicious and have strict requirements for cooking methods. This requires restaurant chefs to plumb and create different treatments to balance the taste and texture of vegetarian food to improve the customer experience. Fourth, vegetarian items should be publicized and labeled. There is a commonly recognized false perception that only Indian restaurants cater to vegetarians because of cultural cognition. However, many types of restaurants already offer vegetarian food. Therefore, they should provide appropriate instructions and promotions for vegetarian options on the menu, such as a conspicuous indication of vegetarian items, and sampling should be utilized as it helps draw attention to vegetarian products and hearten their consumption. Fifth, keep the characteristics of the restaurant while complying with the development of the times. As the transformation of restaurant operations has a certain impact on loyal consumers, restaurant operators should keep their characteristics while keeping up with the trend, especially in food. Finally, a menu with vegetarian and non-vegetarian options should be prepared. Group dining including vegetarians usually selects vegetarian-friendly restaurants that provide vegetarian and non-vegetarian items. Because the diversity of vegetarian types is normalized, a restaurant manager is responsible for accommodating and satisfying these new demands and dining groups to gradually raise customer expectations. In total, the findings of this study show that restaurants that meet such dietary requirements can benefit from improved customer experience levels, thereby improving potential profitability.

Social media websites such as TripAdvisor.com provide vegetarian customers with useful information for identifying vegetarian-friendly restaurants that fulfill their special diet demands. Customers have opportunities to freely share their dining experiences and help each other with their insights by using such websites. In addition, such platforms can provide customer feedback for restaurant managers to improve their ways of fulfilling vegetarian customers. Vegetarian diet advocates (e.g., TripAdvisor.com, European Vegetarian Union, and The Vegan Society) may cooperate and further promote the availability of such platforms among vegetarian consumers and hearten restaurant managers to examine reviews from customers.

### 5.3. Limitations and future research

This current study has some limitations. First, the dataset for this study is collected in London. Therefore, compared with other destinations, the results may be more applicable to the experiences of residents and tourists in London. Future studies should include more plentiful data that is collected from wider regions. Second, although this study inspects the experiences of vegetarian customers, the perspectives of these customers on vegetarian-friendly restaurants can be diverse with personalities, social implications, and professional levels. Future studies should take these factors into account and conduct further research. Third, the types of restaurants are not distinguished in this dataset; therefore, the underlying differences in user-generated content among different restaurant types are not developed. Future studies should pay close attention to which vegetarian options or types of restaurants tend to be the most popular among customers to obtain a comprehensive understanding of restaurant managers’ orientation toward vegetarianism. Fourth, this study explains the customer reviews on only one website. Future studies should exploit user-generated content related to vegetarian customers from other social media or social websites to discern the relative topic significance of fulfilling vegetarian customers among broader reviews (e.g., Yelp). Sixth, considering that there may be consistency deviation in customer-generated content, that is, the star rating published by a customer is inconsistent with the review text. Future research should consider the emotional analysis of customer comments to generate emotional indices, and conduct more in-depth research by associating the emotional indices with customer ratings. Last, studies on the economic contributions of vegetarian guests can also reveal the importance of this usually underestimated market segmentation.

## Data availability statement

The original contributions presented in this study are included in the article/Supplementary material, further inquiries can be directed to the corresponding author.

## Author contributions

XZ and CH performed the research process analysis. XZ wrote the first draft of the manuscript. CH and XB gave additional suggestions. All authors contributed to the manuscript reading, revised, and approved the submitted version, background investigation, experimental design, and method of the study.
