# Supplementary material for: Exploring user-generated content related to vegetarian customers in restaurants: An analysis of online reviews
Source: Front Psychol. 2023 Jan 10;13:1043844. doi: 10.3389/fpsyg.2022.1043844 (PMC9871933; doi:10.3389/fpsyg.2022.1043844)
Supplement: Supplementary file 1 [file Table_1.DOCX]

***Supplementary Material***

# Supplementary Tables

**Supplementary Table.** Identified factors of vegetarians

| Label | Highest Prob | FREX | *Exemplary reviews* |
| --- | --- | --- | --- |
| T16 Value  T18 Friendly staff  T1 Indian food  T14 Bad service  T13 Recommended place  T8 Pleasant experience  T6 Bad experience  T15 Food options  T3 Group dining  T11 Food materials  T9 Reservation  T4 Restaurant view  T7 Gluten free menu  T12 Location  T2 Afternoon tea  T20 Veggie burger  T5 Dessert  T10 Deteriorating experience  T17 Cooking  T19 Atmosphere  T21 Meal experience | good, food, servic, vegetarian, great  love, great, friend, food, recommend  food, indian, london, restaur, best  order, wait, tabl, arriv, ask  place, tri, vegetarian, vegan, can  experi, perfect, beauti, wonder, special  just, better, like, much, don’t  dish, meat, main, fish, starter  one, lunch, menu, vegan, time  chicken, veget, sauc, rice, lamb  tabl, busi, book, even, night  restaur, view, menu, vegetarian, dine  gluten, free, menu, pizza, pasta  locat, london, walk, area, street  tea, afternoon, ask, birthday, sandwich  veggi, burger, breakfast, salad, chees  dessert, daughter, chocol, cream, husband  use, year, food, seem, poor  veget, cook, roast, steak, Sunday  live, fun, music, kitchen, feel  food, meal, restaur, littl, enjoy | good, valu, price, choic, reason  definit, amaz, recommend, thank, fantast  indian, india, dishoom, masala, dosa  minut, bill, hour, wait, min, rude  buffet, your, non, healthi, tri  beauti, superb, impecc, sommeli, pair  think, better, noth, bad, els  fish, tapa, eater, meat, platter  colleagu, favourit, one, branch, today  rice, prawn, duck, squid, dumpl  saturday, earli, reserv, friday, busi  view, floor, tower, brasseri, bridg  gluten, free, coeliac, celiac, pasta  station, covent, garden, conveni, tube  tea, afternoon, sandwich, scone, dietary  breakfast, burger, sausag, toast, egg  chocol, ice, sorbet, mouss, tart  use, sum, dim, tasteless, bland  roast, yorkshir, sunday, gravi, steak  music, fun, entertain, play, cat  littl, enjoy, meal, also, found | 1. *[…]. Very good value for money. Only limited on vegetarian options.* 2. *[…]. The beer was rubbish, […]. Prices were reasonable.* 3. *Andrea was very helpful and attentive during our visit thanks so much! […]* 4. *[…]. Our hostess Jess was lovely and so helpful and Stephan was fantastic! […]* 5. *This was the best Indian food I've ever had in London. […]* 6. *[…]. and I've eaten alot of Indian food! […]* 7. *[…]. We watched as the other tables were served their food and seen to many times without our table being attended to once. […]* 8. *Ive been annoyed in a restaurant before but never as angry at the rude service in this place! […]* 9. *[…] I wanted to try this restuarant as I heard a lot of people saying amazing things about it.* 10. *This is a great place − really cute and quaint. […]* 11. *We dined at Le Gavroche in April and had an extraordinary experience. […]* 12. *Absolutely amazing. We went for lunch and had a wonderfully surprising experience. […]* 13. *I'm a vegetarian− and I hated this place. Basically rubbish on a plate. […]* 14. *[…]. There is no other word to describe my experience of Khan's but Bad. […]* 15. *[…]. It has a large selection of dishes of dips […]* 16. *We chose El Forno because some of us have eaten here before & also for the variety of meals to choose from e.g. Fish , meats & vegetarian .* 17. *Took my team from work for a Christmas lunch, […]* 18. *Took the team of people I work with to this place for lunch − a group of seven. (The group included a vegetarian)* 19. *I ordered a chargrill chicken dish that came with sticky rice and vegetables.* 20. *[…] the consistency of broth. Beef rendang was too dry, the curry Assam fish was nice with the okra and eggplant. […]* 21. *[…]. They take reservations too. […]* 22. *[…]. You may need a reservation because it gets quite busy. […]* 23. *The first thing that strikes you when you visit is the wonderful view you get of the Millennium Bridge, Thames and St Paul's Cathedral. […]* 24. *[…]. We were lucky and seated in the window on the ground floor which suited me perfectly.* 25. *[…]. So lovely as a coeliac to be able to choose anything from the menu. There are vegetarian/vegan/dairy free options too.* 26. *Lovely to be able to eat in a completely gluten free kitchen. […]* 27. *[…]. . Location is just off Covent Garden and a very short walking distance from the tube station. […]* 28. *[…]. It's nearby to West Kensington and West Brompton tube stations, and not far from the Earls Court and Olympia exhibitions centres.* 29. *I arranged afternoon tea as a surprise for my mother […]* 30. *So let me start by saying that before we booked to have an Afternoon tea, […]* 31. *This is how burgers should be. We had the bacon plant (vegetarian) for me and my bfy had the Honest burger. […]* 32. *[…] a veggie burger all washed down with kumquat mojitos. […]* 33. *[…]. For dessert we had severally cream chiboust with strawberries and honeycomb,cardamon custard with saffron oranges, pomegranate and langues de chat and cannelli and camp (coffee) ice cream. […]* 34. *[…]. We followed this with two delicious desserts […]* 35. *My family and I have been coming to Royal China for eighteen years, […]. Sadly after my visit tonight everything is not as it once was. […]* 36. *[…]. Since our last visit, several years ago, the place had become 'poshified' and the real Polish quality and taste had been lost. […]* 37. *[…]. However, the remaining vegetables were not cooked well and the Yorkshire pudding was too hard to eat.* 38. *[…]. The Yorkshire pudding was a little overcooked so was too hard. […]* 39. *[…]. The jazz music in the background makes the*   *atmosphere enveloping and sophisticated […]*   1. *[…]. Very nice concept, cool atmosphere, unique, a must visit when you are in London.* 2. *[…]. We are both long time Vegans, and always looking for great Vegan meals while traveling. Mildred's was a great find. The dumplings are to die for. They are just amazing. […]* 3. *Had a wonderful lunch at Gauthier Soho. A lovely French restaurant. Clean and tastefully decorated, not over the top. […]* |

***Note:*** The order follows the prevalence of topics in reviews. In other words, “Value” factor is most prevalent.
